# NCF4 regulates antigen presentation of cysteine peptides by intracellular oxidative response and restricts activation of autoreactive and arthritogenic T cells

**DOI:** 10.1016/j.redox.2024.103132

**Published:** 2024-03-26

**Authors:** Jing Xu, Chang He, Yongsong Cai, Xipeng Wang, Jidong Yan, Jing Zhang, Fujun Zhang, Vilma Urbonaviciute, Yuanyuan Cheng, Shemin Lu, Rikard Holmdahl

**Affiliations:** a National Joint Engineering Research Center of Biodiagnostics and Biotherapy, and Department of Rheumatology, Second Affiliated Hospital, Xi'an Jiaotong University, Xi'an, Shaanxi, 710004, PR China; b Department of Biochemistry and Molecular Biology, School of Basic Medical Sciences, Xi'an Jiaotong University Health Science Center, Xi'an, Shaanxi, 710061, PR China; c Key Laboratory of Environment and Genes Related to Diseases (Xi'an Jiaotong University), Ministry of Education, Xi'an, Shaanxi, 710061, PR China; d Medical Inflammation Research, Division of Immunology, Dept. of Medical Biochemistry and Biophysics, Karolinska Institute, Stockholm, Sweden; e Department of Human Anatomy, Histology and Embryology, School of Basic Medical Sciences, Xi'an Jiaotong University Health Science Center, 710061, Xi'an, PR China; f Department of Joint Surgery, Xi'an Honghui Hospital, Xi'an Jiaotong University Health Science Center, Xi'an, Shaanxi, 710061, PR China; g Department of Cardiology, The Second Affiliated Hospital, Zhejiang University Schoole of Medicine, Zhejiang, Hangzhou, PR China

**Keywords:** Neutrophil cytosolic factor 4 (NCF4, earlier often denoted p40phox), Neutrophil cytosolic factor 1 (NCF1, earlier often denoted p47phox), Intracellular reactive oxygen species, Redox regulation, Antigen presentation, T cell activation, Arthritis

## Abstract

Autoimmune diseases, such as rheumatoid arthritis (RA) and systemic lupus erythematous, are regulated by polymorphisms in genes contributing to the NOX2 complex. Mutations in both *Ncf1* and *Ncf4* affect development of arthritis in experimental models of RA, but the different regulatory pathways mediated by NOX2-derived reactive oxygen species (ROS) have not yet been clarified.

Here we address the possibility that intracellular ROS, regulated by the NCF4 protein (earlier often denoted p40phox) which interacts with endosomal membranes, could play an important role in the oxidation of cysteine peptides in mononuclear phagocytic cells, thereby regulating antigen presentation and activation of arthritogenic T cells.

To study the role of NCF4 we used mice with an amino acid replacing mutation (NCF4^R58A^), which is known to affect interaction with endosomal membranes, leading to decreased intracellular ROS production. To study the impact of NCF4 on T cell activation, we used the glucose phosphate isomerase peptide GPI_325-339_, which contains two cysteine residues (325-339c-c). Macrophages from mice with the NCF4^58A^ mutation efficiently presented the peptide when the two cysteines were intact and not crosslinked, leading to a strong arthritogenic T cell response. T cell priming occurred in the draining lymph nodes (LNs) within 8 days after immunization. Clodronate treatment, which depletes antigen-presenting mononuclear phagocytes, ameliorated arthritis severity, whereas treatment with FYT720, which traps activated T cells in LNs, prohibited arthritis.

We conclude that NCF4-dependent intracellular ROS maintains cysteine peptides in an oxidized crosslinked state, which prevents presentation of peptides recognized by non-tolerized T cells and thereby protects against autoimmune arthritis.

## 1 Introduction

Rheumatoid arthritis (RA) is a common autoimmune disease with a prevalence of 0.5–1% [1] but there are currently no effective preventive or curative treatments. The cause of this complex disease is unknown, but multiple genetic and environmental factors are likely involved. A neutrophil cytosolic factor 1 (*NCF1*) polymorphic allele, which causes a low reactive oxygen response, has been shown to be a causative factor in SLE and possibly also in RA [[2], [3], [4]]. Associations between RA and single nucleotide polymorphisms (SNP) in the *NCF4* gene have also been identified [2,5]. Both NCF1 and NCF4 are components of the NADPH oxidase type 2 (NOX2) complex, which is known to be responsible for the induced production of reactive oxygen species (ROS) [6]. A mutation of *Ncf1* in the mouse (*Ncf1*^*m1J*^), which causes production of a truncated deficient protein, has been widely used to demonstrate the role of NCF1 in the control of autoimmune disease models. Mice with the *Ncf1*^*m1J*^ mutation are more susceptible to collagen-induced arthritis (CIA) and may also spontaneously develop arthritis during the postpartum period [7]. However, much less is known about the role of NCF4.

An ROS response is initiated by inflammatory signals that activate NCF1, leading to the formation of a complex together with NCF2 and NCF4, which adheres to the membrane flavocytochrome *b*. The NCF1 and NCF4 proteins bind to different types of regulatory membrane lipids. The phox homology (PX) domain of NCF4 binds to the membrane phospholipid phosphatidylinositol 3 phosphate (PI3P), which is normally abundant in the membranes of endosomes, phagosomes, and lysosomes. PI3P enhances NOX2 activity and produces intracellular ROS [8]. A point mutation leading to the replacement of arginine 58 with alanine (R58A) in the PX domain of NCF4 could potentially affect the intracellular ROS production [9]. Mutations of NCF4 in which arginine at position 58 is replaced with cysteine cause a mild, atypical form of chronic granulomatous disease in humans [10]. Our previous work showed that the NCF4^58A^ mutation mediated a lower intracellular ROS and promoted susceptibility to CIA [11], whereas no significant effect on the development of mannan-induced psoriasis (MIP) [12] was found, indicating that NCF1 and NCF4 operate through different mechanisms. It is possible that the preferential ability of NCF4 to promote intracellular, rather than extracellular burst, may play a role in the observed outcomes.

Redox balance is of importance in most biological mechanisms and contributes to both physiological and pathological conditions. Hydrogen peroxide regulates the proliferative signal through ERK activation, while O_2_^−^ enhances a pro-death signal in mature T cells via induction of FasL expression [13]. Changes in cysteine oxidation may alter cellular signaling via the inhibition of tyrosine phosphatases [14], STAT3 [15], and certain MAPK signals [16]. The redox microenvironment within endosomes and phagosomes influences antigen processing and antigenic peptide repertoires [17]. In this study, we used a T cell-dependent mouse arthritis model to investigate the role of intracellular ROS, regulated by NCF4^58A^, in arthritis development.

Glucose-6-phosphate isomerase (GPI) is a ubiquitously expressed protein that participates in glycolysis. K/BxN mice, which express GPI-specific TCR transgenic T cells, were found to develop arthritis [18,19] mediated by cartilage-binding antibodies [20]. Later, GPI was identified as an auto-antigen in RA patients [19], and immunization with GPI protein was found to induce arthritis in DBA/1 mice with a T cell [21] and B cell [22] dependent pattern. Our previous studies showed that induction of arthritis after immunization with the peptide GPI_325-339_ (GIA) in B10Q mice depends on both T cells and B cells but not antibodies [23]. In addition, we found that mice with the *Ncf1*^*m1J*^ mutation could modify T cell responses to cysteine-containing peptides and enhance arthritis [24], suggesting that the lack of ROS could affect T cell activation via antigen processing and presenting cells, such as macrophages. However, the role of NCF4-mediated intracellular ROS in regulating T cell activation remains an open question.

In the present study we show that the NCF4^58A^ mutation leads to severe arthritis by promoting T cell proliferation and activation. Notably, T cells themselves did not express a detectable level of NOX2 complex proteins or produce NOX2 complex-driven ROS. The lower ROS production in NCF4^58A^ antigen-presenting cells (APCs) prevented crosslinking of the cysteines in the GPI_325-339_ peptide, allowing the peptide to be presented to T cells and activate them. This T cell activation occurred in draining LNs before day 8 after antigen immunization. Subsequently, the primed T cells migrated out of LNs to peripheral tissue, including joints. We observed that trapping the activated T cell via FTY720, or deleting APCs (including macrophages) via clodronate, could alleviate arthritis development. Taken together, our findings show that NCF4-mediated intracellular ROS in APCs affect antigen processing of the GPI_325-339_ peptide and prevent activation of arthritogenic T cells, thus providing protection from arthritis.

## 2 Results

### 2.1 The NCF4^58A^ mutation accelerates GIA

**Fig. 1:**
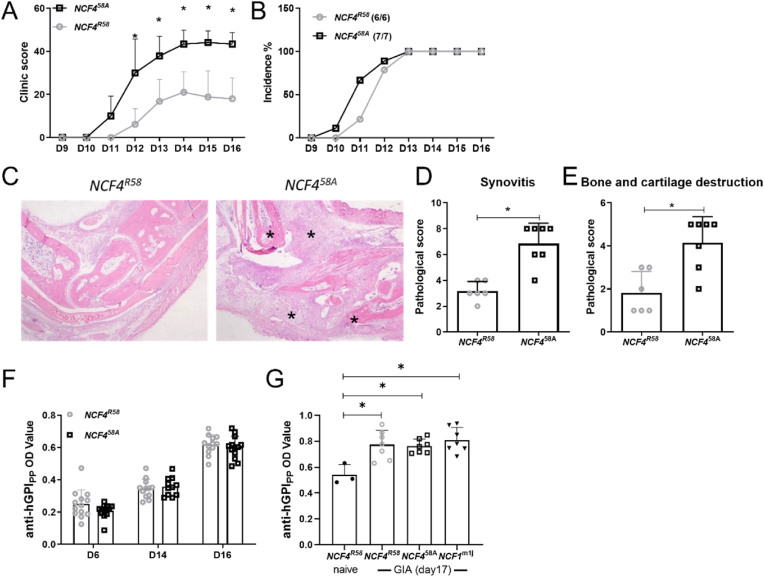
GIA is exacerbated by the NCF4^58A^ mutation in B10Q mice. The macroscopic arthritis scores (A) and the incidence of arthritis (B) were compared between groups (multiple *t*-test with Holm-Sidak's comparison correction). Representative histological images of ankle joints stained by H&E (C), * mark the inflamed affected area of synovia and cartilage. The histologic scores of synovitis (D) and bone and cartilage destruction (E) were quantified for both groups (two-tailed Mann–Whitney *U* test), (*NCF4*^*R58*^ n = 6, *NCF4*^*58A*^ n = 7). The anti-GPI_325-339_ antibody titers were determined on day 6, day 14, day 16 (F) and day17(G) (two-tailed Mann–Whitney *U* test), (*NCF4*^*R58*^ n = 13, *NCF4*^*58A*^ n = 12 or *NCF4*^*R58*^ n = 3(naive)/n = 7, *NCF4*^*58A*^ n = 7 and *NCF1*^*m1J*^ n = 7).

To investigate the role of NCF4^58A^ in regulating immune response and arthritis development, GPI_325-339_ peptide was used to induce arthritis in littermate B10Q mice expressing NCF4^58A^ or the wildtype (WT) NCF4^R58^ allele. Mice with the NCF1^m1J^ mutation were used as a positive control. The NCF4^58A^ mice developed an earlier and more severe arthritis compared to the WT mice (Fig. 1A and B). The histology of ankle joint sections showed more severe synovitis, with more bone and cartilage damage, in NCF4^58A^ mice (Fig. 1C–E). Serum antibodies to the GPI_325-339_ peptide were detectable, but, as expected, at very low levels, in all groups, with no difference between the two NCF4 alleles (Fig. 1F and G). These results suggest that the NCF4^58A^ mutation leads to an accelerated arthritis induced by the GPI_325-339_ peptide, without influencing the levels of the minimal antibody response.

### 2.2 T cells in draining LNs on day 7 cause arthritis

**Fig. 2:**
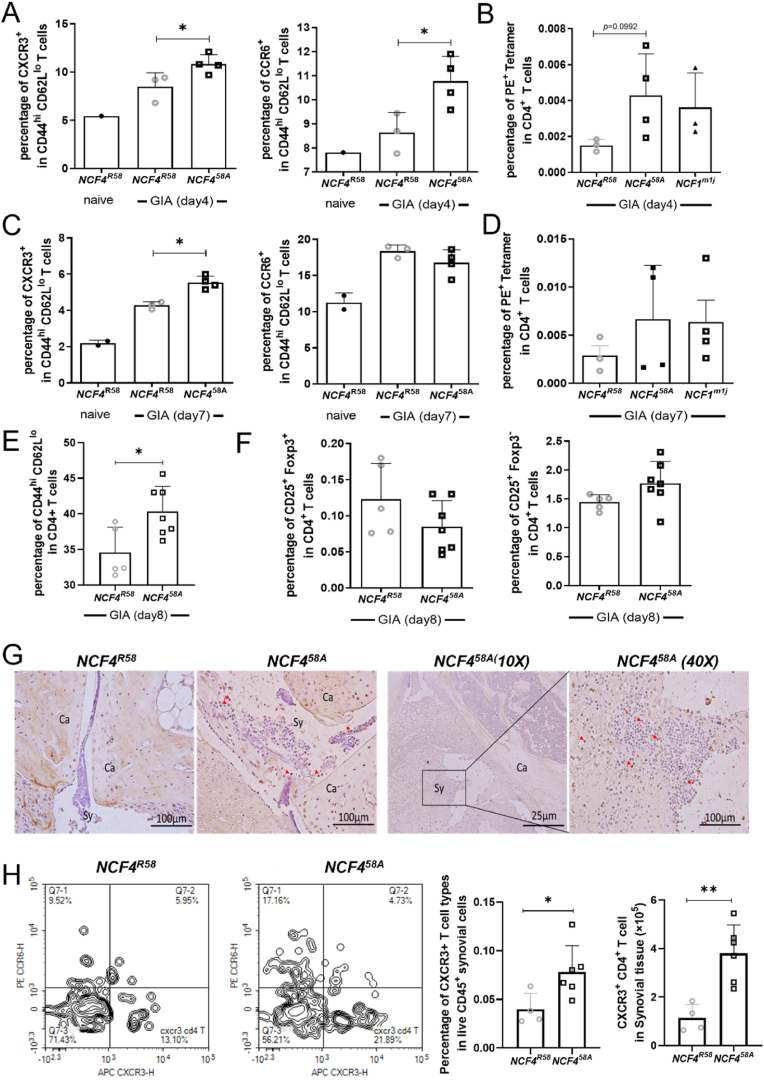
NCF4^58A^ mutation increases the frequency of activated T cells in iLN and synovia. The CXCR3^+^ or CCR6^+^ activated T cells (A, C) and PE Tetramer^+^ in CD4^+^ T cells (B, D) in iLN at day 4 and day 7 in GPI peptide-immunized mice (two-tailed Mann–Whitney *U* test), (*NCF4*^*R58*^ n = 3 or 4, *NCF4*^*58A*^ n = 4). The frequency of CD44^hi^CD62L^lo^ T cells in iLN at day 8 in GPI peptide-immunized mice (E–F) (two-tailed Mann–Whitney *U* test), (*NCF4*^*R58*^ n = 5, *NCF4*^*58A*^ n = 7). Representative CXCR3 positive cells stained by IHC (G) (two-tailed Mann–Whitney *U* test). The percentage and numbers of CXCR3^+^ T cells in synovial tissue (H) compared between groups (*NCF4*^*R58*^ n = 4, *NCF4*^*58A*^ n = 6).

To assess the contribution of immune cells in draining inguinal lymph nodes (iLN) to the development of arthritis, iLN from GPI_325-339_ peptide-immunized mice were eviscerated at different time points before the onset of arthritis. On day 4, both Th1(CXCR3^+^) and Th17(CCR6^+^) cells increased in frequency (Fig. 2A); a trend toward increased frequency of GPI-specific T cells in the NCF4^58A^ mice compared to WT was also observed (Fig. 2B). On day 7, only Th1 cells, but not Th17 or GPI-specific T cells, were still increased in NCF4^58A^ mice as compared with WT mice (Fig. 2C). No difference was found in the frequency of GPI-specific T cells between the two groups of mice (Fig. 2D). On day 8, we observed a higher frequency of activated T cells (CD44^hi^CD62L^lo^) in iLN from NCF4^58A^ mice compared to WT mice (Fig. 2 E), but with no difference in Treg cell frequencies (Fig. 2F). Finally, CXCR3 positive cells were also found in synovial tissues from arthritic joints at day 17 (Fig. 2G), and more CXCR3^+^ T cells were found in synovia of NCF4^58A^ mice than in WT mice (Fig. 2H). Thus, the activation of T cells in the draining iLN could be a critical step in the development of arthritis.

**Fig. 3:**
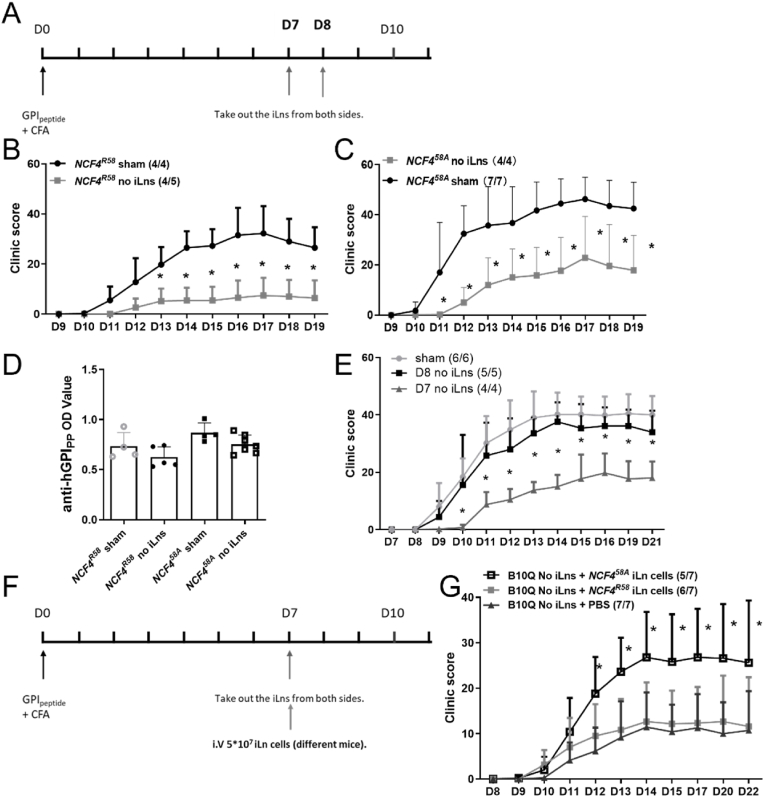
Lymphocytes in iLN, 7 days after immunization of *Ncf4*^*58A*^ mice, cause arthritis. Mice were immunized with GPI peptide and iLN were excised after different time periods (A). Arthritis scores of immunized *Ncf4*^*R58*^ (B) and *Ncf4*^*58A*^ mice (C) before and after iLN removal (multiple *t*-test with Holm-Sidak's comparison correction). The anti-GPI_325-339_ antibody titer determined in sera obtained at day 19 (D) (*NCF4*^*R58*^ n = 9, *NCF4*^*58A*^ n = 11). The macroscopic arthritis scores of immunized mice with iLN removed on day 7, day 8, or with sham excision were compared among groups (E) (multiple *t*-test with Holm-Sidak's comparison correction), (B10Q n = 16). The scheme of the iLN cells transfer experiment (F). The macroscopic arthritis scores of recipient mice with NCF4^R58^, NCF4^58A^ iLN cell reinjection or with PBS compared between groups (G) (multiple *t*-test with Holm-Sidak's comparison correction) (B10Q n = 7, NCF4^R58^ n = 7, NCF4^58A^ n = 7).

Removing the iLNs on day 7 prohibited the development of arthritis in both WT and NCF4^58A^ mice (Fig. 3A–C) but did not affect the titer of anti-GPI_325-339_ antibodies (Fig. 3D). Interestingly, removal of iLNs on day 8 did not alleviate arthritis (Fig. 3E), indicating that the arthritogenic T cells had already migrated to joints. To confirm that T cells in the draining LNs were primed to be arthritogenic, we isolated lymphocytes from iLNs of NCF4^58A^ or WT on day 7, and then transferred them to WT mice that had previously undergone iLN excision (Fig. 3F). The iLN cells from NCF4^58A^ mice induced severe arthritis (Fig. 3G), more severe than with WT donors, indicating that the lower ROS response by NCF4^58A^ led to the accumulation of primed arthritogenic T cells at day 7. These results suggest that T cells are activated in the draining LNs during the first week after immunization but leave before day 8 to mediate arthritis. The induced arthritis was more severe if the T cells were derived from NCF4^58A^ mice. We conclude that the T cells are primed in the draining LNs before day 8 and thereafter migrate to the joints to induce arthritis, and that NCF4^58A^ enhanced their arthritogenicity.

### 2.3 The NCF4^58A^ mutation promotes T cell activation in GIA

**Fig. 4:**
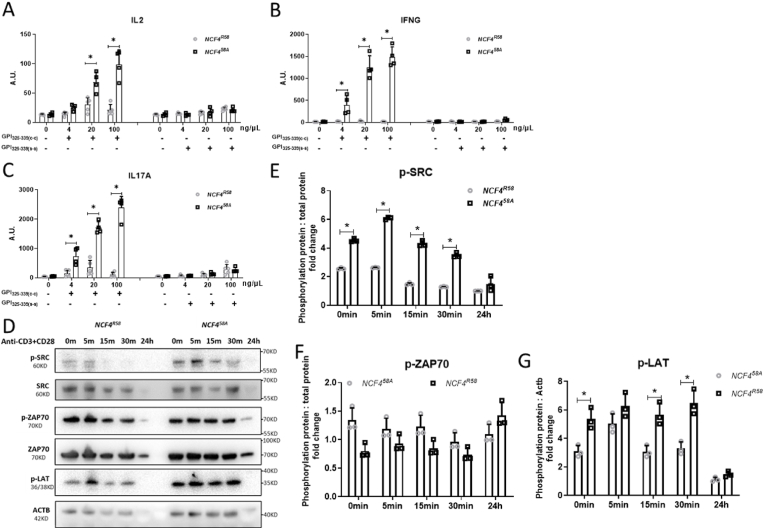
T cells from NCF4^58A^ mutated mice show increased proliferation and activation after GPI_325-339_ priming. The cytokine secretion of IL2 (A), IFNγ (B), and IL17A (C) from iLN 7 days after GPI peptide immunization of NCF4^R58^ and NCF4^58A^ mice that were stimulated with GPI_325-339_ c-c or s-s peptides. (IL2 detection after 24 h incubation and IFNγ and IL17A detection after 72 h incubation) (Kruskal–Wallis test with Dunn's *post hoc* test) (NCF4^R58^ n = 4, Ncf4^58A^ n = 4). Isolated T cells from iLN of NCF4^R58^ and NCF4^58A^ mice 7 days after GPI immunization were treated with anti-CD3 and anti-CD28 for 5, 15, 30 min or 24 h and blotted with *p*-SRC, SRC, p-ZAP70, ZAP70, *p*-LAT and β-actin (ACTB) antibodies (D). The phosphorylated SRC or ZAP70 was analyzed by comparing levels with total protein, and LAT was analyzed by comparison with ACTB (E–G) (two-tailed Mann–Whitney *U* test) (NCF4^R58^ n = 3, NCF4^58A^ n = 3).

To examine the impact of NCF4^58A^ on T cell function, we conducted an immune response recall assay to the GPI_325-339_ peptide, which contains two cysteines. The GPI peptide is critical for TCR recognition but can potentially be oxidized in APCs [23,24]. Thus, we tested both the standard peptide with reduced cysteines at positions 330 and 333 (c-c) and the oxidation-insensitive peptide with serines replacing the cysteines at the same positions (s-s). The draining LN cells obtained at 7 days after GPI_325-339_(c-c) immunization were stimulated with either c-c or s-s peptide. After 72 h, the supernatants were collected and quantified by ELISA for IL2, IFNγ, and IL17. LN cells from NCF4^58A^ mice stimulated with the c-c peptide produced higher amounts of IL2 (Fig. 4A), IFNγ (Fig. 4B), and IL17 (Fig. 4C) as compared with LN cells from WT mice. These data suggest that the decreased intracellular ROS due to the NCF4^58A^ allele allowed a more pronounced T cell activation and proliferation response.

To investigate how NCF4-dependent ROS influenced T cell activation, T cells were isolated from the draining iLNs of GPI_325-339_-immunized mice on day 7. After stimulation of these T cells with CD3 and CD28, we found that the T cells from NCF4^58A^ mice became activated earlier than T cells from WT mice (Fig. 4D–G). In particular, linker for activation of T cells (LAT) and proto-oncogene tyrosine kinase sarcoma (SRC; c-SRC) were more phosphorylated in T cells from the NCF4^58A^ mutated mice. In fact, the T cells from NCF4^58A^ mice were more activated even before the addition of CD3/CD28 antibodies, showing that these T cells were already activated *in vivo*. Thus, antigen presentation activates T cells more efficiently in NCF4^58A^ mutant mice.

### 2.4 The NOX2 complex is expressed in macrophages rather than T cells

**Fig. 5:**
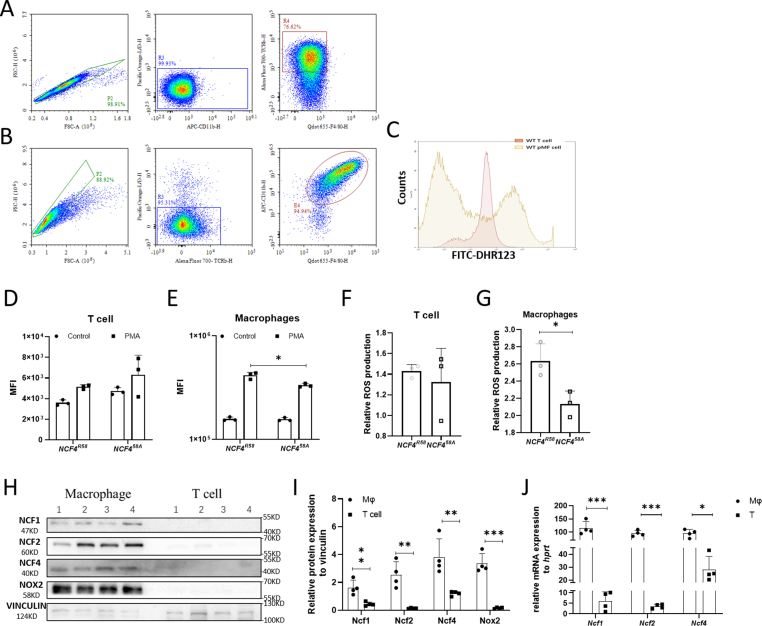
NOX2 complex components are mainly expressed in macrophages not in T cells. The purity of MACS-sorted T cells and macrophages (A–B). Representative plots for the ROS production of PMA-stimulated macrophages (reddish) and T cells (yellowish) are shown in a histogram (C). The ROS production of PMA-stimulated and unstimulated T cells (D) and macrophages (E). The relative ROS production of NCF4^R58^ and NCF4^58A^ T cells (F) and macrophages (G) (two-tailed Mann–Whitney *U* test) (NCF4^R58^ n = 3, NCF4^58A^ n = 3). Isolated T cells and macrophages were blotted with NCF1, NCF2, NCF4, NOX2 and VINCULIN antibodies (H). The protein (I) (two-tailed Mann–Whitney *U* test) and mRNA (J) (one way ANOVA, unpaired Student t-test) expression of NCF1, NCF2, NCF4 and NOX2 (NCF4^R58^ n = 4, NCF4^58A^ n = 4).

To verify if NOX2-derived ROS intrinsically regulated T cell function, T cells and macrophages were isolated and investigated for expression of the major components of the NOX2 complex. We isolated untouched T cells and macrophages from naïve mice (Fig. 5A and B). After PMA stimulation, macrophages from WT mice induced a high DHR-123 fluorescence intensity (10 [6]), whereas the response in T cells was indistinguishable from background (10 [3]). Macrophages from NCF4^58A^ mice had a lower ROS response than macrophages from WT mice, whereas no differences were seen in T cells (Fig. 5C–G). The data were confirmed by RNA and protein expression analyses of NCF1, NCF2, NCF4 and NOX2 (Fig. 5H–J). Therefore, our findings suggest that NCF4 intrinsically regulates APCs, such as macrophages, and only indirectly regulates T cell function.

### 2.5 Macrophages with the NCF4^58A^ mutation, with low intracellular ROS, allow a stronger pro-inflammatory cytokine response determined by increased STAT1 and P65 phosphorylation

**Fig. 6:**
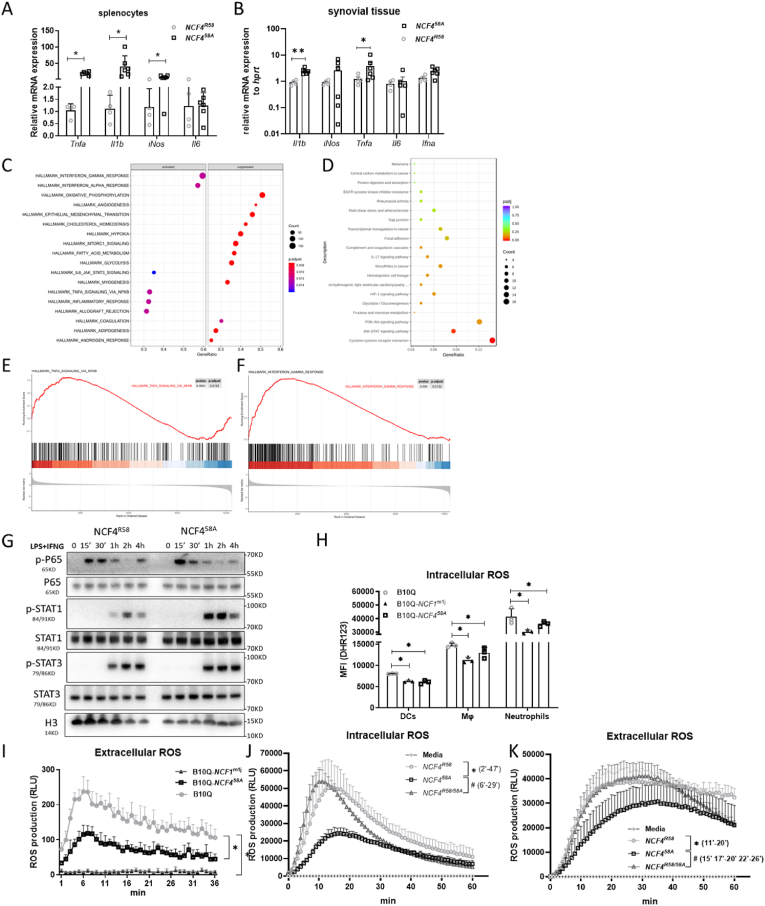
NCF4^58A^ mutation increases macrophage cytokine expression associated with P65 and STAT1 activation and affects intracellular ROS production. The mRNA expression of *Tnfα, Il1β, iNos* and *Il6* in spleen cells (A) and synovial cells (B) isolated 17 days after GPI peptide immunization (one way ANOVA, unpaired Student t-test) (NCF4^R58^ n = 4, NCF4^58A^ n = 6). The GSEA (C), GO (D) analysis and pathway enrichment (E–F) of classical activated BMMs from NCF4^R58^ and NCF4^58A^. (NCF4^R58^ n = 3, NCF4^58A^ n = 3). The classical activated BMMs from Ncf4^R58^ and NCF4^58A^ blotted via p-P65, P65, p-STAT1, STAT1, p-STAT3, STAT3 and H2B antibodies (H). The relative intracellular ROS production of DCs (CD11c^+^), macrophages (CD11b^+^F4/80^+^), and neutrophils (CD11b^+^Ly6G^+^) from the spleens of B10Q, *NCF1*^*m1J*^ and *NCF4*^*58A*^ mice (H) (two-tailed Mann–Whitney *U* test). Extracellular ROS of splenocytes from B10Q, *NCF1*^*m1J*^ and *NCF4*^*58A*^ mice (I) (multiple *t*-test with Holm-Sidak's comparison correction) (B10Q n = 3, *NCF1*^*m1J*^ n = 3, *NCF4*^*58A*^ n = 3). The intracellular (J) and extracellular (K) ROS production of macrophages from *NCF4*^*R58*^*, NCF4*^*R58/58A*^ and NCF4^58A^ mice (multiple *t*-test with Holm-Sidak's comparison correction) (*NCF4*^*R58*^ n = 3, *NCF4*^*R58/58A*^ n = 4, *NCF4*^*58A*^ n = 4).

A strong proinflammatory cytokine response was still seen at day 17 after GPI peptide immunization in splenocytes from NCF4^58A^, but not WT NCF4^R58^ mice; mRNA expression of *Tnfa*, *Il1b*, *iNos* were up-regulated, while *Il6* showed no differences between the 2 genotypes (Fig. 6A). Additionally, mRNA expression of *Tnfa*, *Il1b* from day17 in synovial tissue were also up-regulated in cells from NCF4^58A^ compared to WT (Fig. 6B). RNA-seq was performed to identify the gene expression pattern regulated by NCF4^R58A^ in activated macrophages. Gene set enrichment analysis (GSEA) (Fig. 6C) and gene ontology (GO) analysis (Fig. 6D) showed enrichment of IFNG and TNFA cytokine signal-associated genes in the NCF4^58A^ macrophages compared with the WT macrophages (Fig. 6E and F). To verify these results, we examined the activation of key transcription factors in the IFNG and TNFA pathways. The results indicated enhanced phosphorylation of P65 (NFκB) and STAT1 in NCF4^58A^ macrophages (Fig. 6G).

NCF4, in contrast to NCF1, is known to preferentially activate the NOX2 complexes at endosomal membranes rather than at cellular membranes, and our previous results showed that the presence of WT NCF4 leads to a higher intracellular ROS response in neutrophils [11]. To determine the role of NCF4 in macrophages we compared the intracellular and extracellular ROS in splenocytes from *Ncf4*^*R58*^*, Ncf4*^*58A*^ and *Ncf1*^*m1J*^ mice. Compared with WT mice, the intracellular ROS in dendritic cells (DCs), macrophages, and neutrophils were decreased in NCF4^58A^ and NCF1-deficient mice (Fig. 6H). The extracellular ROS response almost vanished in NCF1-deficient cells, whereas only a slight decrease was found in NCF4^58A^ cells (Fig. 6I). Comparing intracellular and extracellular ROS in WT and NCF4^58A^ macrophages, we found that homozygous (NCF4^58A^) macrophages showed a decreased intracellular ROS production compared to WT macrophages (Fig. 6J). Cells with homozygous NCF4^58A^ showed a milder effect on extracellular ROS response compared to cells with heterozygous NCF4^R58/58A^ (Fig. 6K).

Having shown that NOX2 complex components are predominantly expressed in macrophages rather than T cells, we next asked how the NCF4^58A^ mutation, which is expressed in antigen-presenting macrophages with a decreased intracellular ROS induction capacity, could affect T cell activation and arthritis development.

### 2.6 NCF4^58A^ affects antigen presentation of GPI_325-339_ peptides

**Fig. 7:**
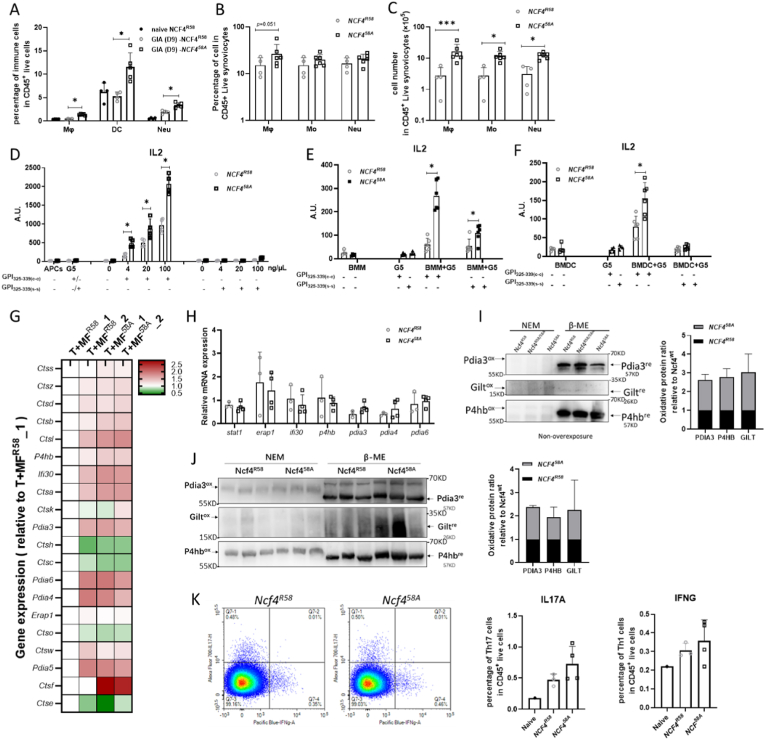
The NCF4^58A^ mutation affects antigen presentation of cysteine-cysteine GPI_325-339_ peptides. The relative frequencies of macrophages (CD11b^+^F4/80^+^), DCs (CD11c^+^MHCII^+^) and neutrophils (CD11b^+^Ly6G^+^) in spleens 7 days after GPI peptide immunization of NCF4^R58^ and NCF4^58A^ mice (A) (one way ANOVA, unpaired Student *t*-test) (NCF4^R58^ n = 8, NCF4^58A^ n = 5). The relative frequencies (B) and cell numbers (C) of macrophages, monocytes (CD11b^+^Ly6C^+/hi^), and neutrophils in synovial tissue obtained 17 days after GPI peptide immunization of NCF4^R58^ and NCF4^58A^ mice (one way ANOVA, unpaired Student t-test) (NCF4^R58^ n = 4, NCF4^58A^ n = 6). IL2 from G5 T hybridoma cells determined by co-culturing with splenocytes from GPI peptide-immunized mice (D), naïve BMMs (E) or naïve BMDCs (F) together with GPI_325-339_ c-c or s-s (NCF4^R58^ n = 4, NCF4^58A^ n = 4) (Kruskal–Wallis test with Dunn's post hoc test). The expression of antigen processing-related genes analyzed in NCF4^R58^ and NCF4^58A^ macrophages co-cultured with GPI_325-339_ peptide and naïve T cells (G) (NCF4^R58^ n = 2, NCF4^58A^ n = 2). The mRNA expression of *Stat1, Erap1, Ifi30, P4hb, Pdia3, Pdia4* and *Pdia6* analyzed in splenocytes obtained 17 days after GPI peptide immunization (H) (two-tailed Mann–Whitney *U* test). Both non-reduced (NEM) and reduced (β-ME) forms of the thiol group in Pdia3, Gilt and P4hb detected in splenocytes obtained 17 days after GPI peptide immunization (I) (NCF4^R58^ n = 3, NCF4^58A^ n = 3). Both non-reduced (NEM) and reduced (β-ME) forms of the thiol group in PDIA3, GILT and P4HB detected in splenocytes obtained 7 days after GPI peptide immunization (J) (two-tailed Mann–Whitney *U* test) (NCF4^R58^ n = 3, NCF4^58A^ n = 3). IL17A and IFNγ positive CD4^+^ T cell (K) determined in iLN obtained 7 days after immunization with collagen type II (two-tailed Mann–Whitney *U* test) (NCF4^R58^ n = 3, NCF4^58A^ n = 4).

To investigate if NCF4^R58A^ affects antigen presentation, we first determined the effects on the number of antigen-presenting mononuclear phagocytes, including macrophages and DCs. In GPI_325-339_-immunized WT and NCF4^58A^ mice, we found that the proportion of DCs, macrophages and neutrophils (Fig. 7A) were increased at day 9 in the spleens of NCF4^58A^ mice compared to the spleens of WT mice. The proportions and total numbers of potential antigen-presenting phagocytic cells in synovial tissue of arthritic joints at day 17 were determined in WT and NCF4^58A^ mice (Fig. 7B). Macrophages, monocytes, and neutrophils were increased in NCF4^58A^ mice compared to WT mice (Fig. 7C). To assess the antigen presentation efficiency, naive bone marrow-derived macrophages (BMMs), bone marrow-derived DCs (BMDCs), and splenocytes isolated from mice immunized 7 days previously were co-cultured with GPI_325-339_ specific T cell hybridoma cells (G5) to test the presentation of GPI_325-339_ c-c and s-s peptides. Crosslinking of the cysteines is known to prohibit binding to MHCII due to changed peptide structure, although both positions are directed to the T cell receptor [24]. NCF4^58A^-expressing splenocytes were more efficient in presenting the c-c peptide to G5 cells than the WT cells, while no responses were seen with the s-s peptides (Fig. 7D). These data indicate that the lower ROS response in NCF4^58A^ cells reduced at least some of the peptides, allowing binding to the MHCII. The same results were also found with BMMs (Fig. 7E) and BMDCs (Fig. 7F), suggesting that the NCF4^58A^-dependent intracellular ROS affects the GPI_325-339_ peptide antigen presentation process in APCs.

To further explore this finding, GPI_325-339_ peptide-treated macrophages were analyzed with RNA-seq. Antigen presentation-related gene expression was slightly increased in NCF4^58A^ macrophages (Fig. 7G). No significant differences were found in the mRNA levels of the antigen presentation-related genes *Stat1, Erap1, P4hb, Pdia3, Pdia4, Pdia6* and *Ifi30* in NCF4^58A^ splenocytes (Fig. 7H). To investigate whether NCF4-dependent intracellular ROS affects thiols of these antigen presentation-related proteins, we conducted redox Western blotting analyses on proteins of splenocytes from GPI_325-339_ or collagen type II immunized WT and NCF4^58A^ mice. The reduced forms of PDIA3, GILT and P4HB were significantly increased in GIA (Fig. 7I) and CIA (Fig. 7J) NCF4^58A^ splenocytes, suggesting that oxidation was involved in the regulation of antigen processing protein modification and activity. However, both Th1 and Th17 cells showed no significant increase in CIA in day 7 iLN (Fig. 7K). These results suggest that intracellular ROS controlled by NCF4 regulated antigen processing by modifying antigen processing proteins as well as regulating SS crosslinking of the antigenic peptide. The diminished intracellular ROS, due to the NCF4^R58A^ mutation, enabled APCs to efficiently present the immunodominant GPI peptide in its reduced form.

### 2.7 Deletion of mononuclear phagocytes and trapping T cells in iLNs alleviates arthritis development

**Fig. 8:**
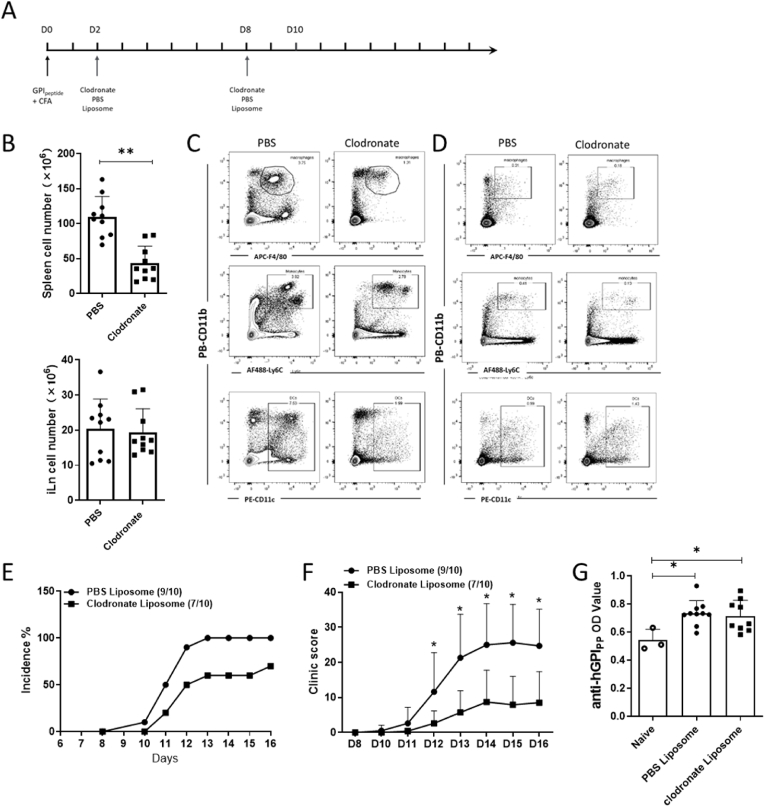
Deletion of mononuclear phagocytes via clodronate liposome treatment alleviates GIA. The scheme of mononuclear phagocyte deletion in mice immunized with GPI peptide (A). The cell number of splenocytes and iLNs at day 17 after treatment of GPI peptide-immunized mice with PBS or clodronate (B) (two-tailed Mann–Whitney *U* test). The proportion of macrophages (CD11b^+^F4/80^+^), monocytes (CD11b^+^Ly6C^+/hi^) and DCs (CD11c^+^) among splenocytes (C) and blood (D) at day 17 after GPI peptide immunization after PBS or clodronate treatment. The incidence of arthritis (E) and the macroscopic arthritis scores (F) compared between groups (multiple *t*-test with Holm-Sidak's comparison correction). The anti-GPI_325-339_ antibody titers in sera obtained day 17 after GPI peptide immunization (G) (two-tailed Mann–Whitney *U* test) (B10Q n = 10).

To verify the role of APCs in arthritis development, we deleted them with clodronate liposome treatment. On day 2 and day 8 after immunization, we injected clodronate or PBS liposomes intravenously (Fig. 8A). The number of splenocytes declined in clodronate-treated mice, while no effects were seen on the number of iLN cells (Fig. 8B). Numbers of splenic mononuclear phagocytes, including macrophages, monocytes and DCs, decreased after clodronate injection (Fig. 8C), and we also noted a loss of monocytes in blood (Fig. 8D). The incidence of arthritis and arthritis severity decreased after clodronate treatment (Fig. 8E and F). However, clodronate did not influence the low levels of antibodies to the GPI peptide (Fig. 8G), confirming that the pathogenicity is dependent on mononuclear phagocytes and T cells rather than antibodies.

**Fig. 9:**
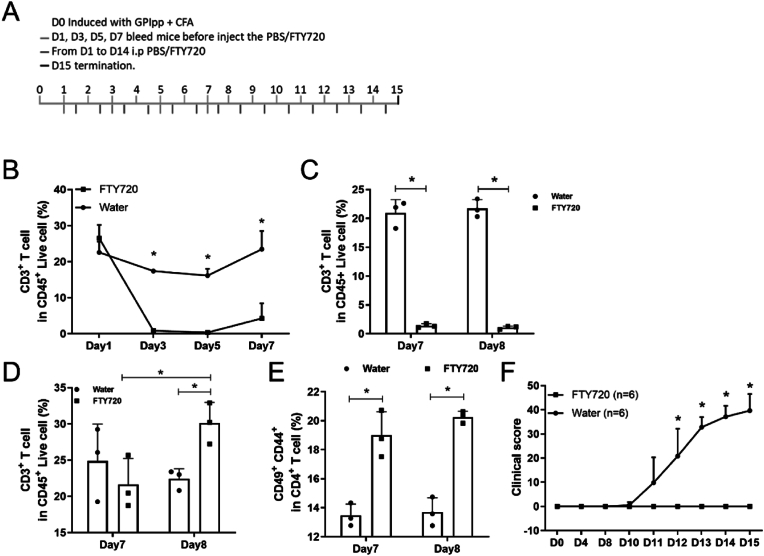
Trapping of activated T cells in LN by FTY720 treatment ameliorates GIA. The scheme of FTY720 treatment in GIA development (A). The proportion of T cells in blood followed on day 1, day 3, day 5, day 7 (B) after GPI_325-339_ peptides immunization (one way ANOVA, unpaired Student t-test). The proportion of T cells in blood (C) and iLNs (D) 7 and 8 days after GPI peptide immunization (one way ANOVA, unpaired Student t-test). The frequency of activated T cells in iLNs (E) 7 and 8 days after GPI peptide immunization (one way ANOVA, unpaired Student t-test). The macroscopic arthritis scores (F) were compared between groups (multiple *t*-test with Holm-Sidak's comparison correction) (B10Q n = 6).

To investigate the role of T cells in arthritis development, we trapped the activated T cells in the draining LNs by blocking chemotaxis function through treatment with FTY720, an agonist of the sphingosine 1-phosphate (S1P) receptor [25,26]. By injecting this drug daily intraperitoneally after immunization (Fig. 9A), we found that the frequencies of CD3^+^ T cells in CD45^+^ live cells in blood decreased from day 3 and remained at a low frequency until day 8 (Fig. 9B). The relative numbers of CD3^+^ T cells (among CD45^+^ cells) in the blood were at a very low level in FTY720-treated mice on day 7 and day 8 (Fig. 9C). During the same time, the frequency of T cells increased from day 8 (Fig. 9D), and the activated T cells were successfully trapped in the iLN on day 7 and day 8 (Fig. 9E). During prolonged treatments with FTY720, the signs of arthritis were completely suppressed (Fig. 9F). These results suggest that removing APCs or trapping T cells in LNs protects mice from arthritis.

## 3 Discussion

We show that a mutation that hinders NCF4 interactions with endosomal membranes enhances antigen processing and presentation of cysteine-containing peptides, thereby allowing activation of T cells in the draining LNs and increasing arthritis susceptibility. Due to presentation of reduced GPI_325-339_ cysteine peptide by mononuclear phagocytes, such as macrophages, T cells in draining LNs are activated within the first 7 days after immunization. This activation is controlled by intracellular ROS regulated by NCF4 in mononuclear phagocytes.

The immune regulatory role of the NOX2 complex was first discovered by the positional cloning of the NCF1 polymorphism associated with autoimmune arthritis [27]. The NOX2-derived ROS was found to oxidize the interacting T cells during antigen presentation by exposing the T cell to ROS during antigen presentation [28,29]. However, we later identified another parallel mechanism, which involved redox regulation of cysteine-containing peptides within the APCs [24]. In a previous report we found that mice carrying the *Ncf1*^*m1J*^ mutation developed more severe arthritis due to antigen presentation of the reduced cysteine peptide to poorly tolerized T cells [24]. The *Ncf1*^*m1J*^ mutation leads to expression of only small amounts of deficient NCF1 protein, abrogating both intracellular and extracellular ROS response by the NOX2 complex [11,30]. We also showed that mice with the NCF4^58A^ mutation developed severe CIA due to dysregulated B cells and lost the ability to regulate plasma cell differentiation [31]. These data suggested that NCF4 and NCF1 regulate autoimmunity and the development of arthritis differentially. The NCF4 N-terminus contains a PX domain, which has a high specificity for the phospholipid PI3P, as proven by crystallization and X-ray diffraction [32,33]. PI3P is generated during phagocytosis in the phagosomal membrane and on early endosomes by the Class III PI3 kinase Vps34 [[34], [35], [36]], which is constitutively active on Rab5-positive early endosomes, and the fusion of these endosomes with the nascent phagosome leads to generation of PI3P in the phagosomal membrane [33,37]. However, in the resting state of a phagocyte, NCF4 is unable to bind to PI3P [38]. The binding of PI3P to NCF4 is critical for its positive regulation of NOX2 when assembled on intracellular membranes such as endosomes and phagosomes [39]. The R58A point mutation on NCF4 affects PI3P binding [33], leading to a decreased capacity to produce ROS intracellularly. If the NOX2 complex assembles on the cellular membrane it will mainly affect the interacting T cells through ROS, or most likely through the more stable hydrogen peroxide. On the other hand, if NOX2 complex activity is impacted preferentially on endosomal membranes, it is more likely to only affect cell-intrinsic functions in APCs, including antigen processing. The use of a peptide with two cysteines highlights the importance of NOX2 activation on endosomal membranes, as it could directly regulate antigen presentation by oxidizing the cysteines in the peptide and thereby prevent peptide presentation [24]. Here we show that an amino acid replacing mutation (R58A) on NCF4, which is important for regulating NOX2 activity on endosomal membranes, predominantly decreases the capacity for an intracellular ROS burst in macrophages and other APCs. However, we have not yet addressed the specificity of the intracellular ROS response that mediates oxidation of antigenic peptides and modifies antigen processing [40].

In this study, NCF4 regulated the priming and activation of arthritogenic T cells in the draining LNs prior to day 8 after peptide immunization. In the NCF4^58A^ mice, the T cells had already at day 4 differentiated to express CXCR3 or CCR6, but at day 7 only CXCR3-positive T cells were still increased. In NCF4^R58A^ mice, the T cells exited after day 7 and most likely migrated to the joints to induce arthritis. CXCR3 is a phenotypic marker of Th1 cells and has been found in the joints of RA patients [41]. It has also been reported that an antagonist of CXCR3 suppressed CIA by decreasing the percentage of Th1 and Th17 cells in joints [42].

We observed that T cells isolated from NCF4^58A^ mice at day 7 after GPI peptide immunization had a more pronounced expression of activated SRC and LAT, and we also found these T cells to be more activated after anti-CD3 and anti-CD28 treatment. It is known that LAT phosphorylation leads to the recruitment and activation of phospholipase C-γ1 (PLC-γ1), which decreases the threshold of T cell activation to weaker stimuli, including weak agonists and self-peptides [43]. This phenomenon may explain why the NCF4^58A^ mice had more activated T cells and developed more severe arthritis than WT mice.

Interestingly, the decreased ability to produce endosomal ROS in NCF4^58A^-expressing cells allowed antigen processing GILT, PDIA3, and P4HB protein reduction, with increased thiols. PDIA3 acts directly on antigen peptide redox regulation [44], which is regulated via intracellular ROS. Proteome-wide analysis also showed that P4HB is very sensitive to a redox modified environment [45]. In contrast to the cysteine-containing GPI peptide that causes GIA, we have previously shown that the major peptide from type II collagen, which is of crucial importance for the induction of CIA, was not regulated by NCF4 [11]. These results confirm that the occurrence of cysteines in the antigenic peptide is of crucial importance for NCF4^R58^-mediated regulation. The GPI peptide, which contained two cysteines, is not able to bind the MHCII molecule if it is oxidized and thereby crosslinked, which likely occurs more often in the WT as compared with the NCF4^58A^ mice [46].

There are multiple steps in antigen processing and presentation in the endolysosomal compartments of APCs. The complete loss of function of the *Ncf1*^*m1J*^ mutation leads to an increased GILT expression in macrophages, promoting the reduction of disulfide bonds in the GPI peptide [24]. Here, we report that the NCF4^58A^ point mutation-dependent decrease of intracellular ROS also increased T cell activation via antigen presentation, not necessarily because of GILT overexpression but likely due to the thiol modification on cysteine amino acids. The loss of the enzyme-activated center of the GILT protein via point mutation on cysteines affected the generation of MHCII-restricted CD4^+^ T cell responses to protein antigens with a disulfide bond in the CXXC motif [47]. Disulfide reduction has been shown to occur in the absence of GILT, suggesting the presence of other reductive mechanisms of antigen process [48]. The disparity between *Ncf1*^*m1J*^ and *Ncf4*^*58A*^ mice in antigen presentation could be due to the different levels of intracellular ROS involved in gene transcriptional regulation and protein modification. We also showed a similar modification of P4HB and PDIA3 in WT compared with NCF4^58A^ mice. P4HB catalyzes the formation of 4-hydroxyproline in collagen [49] while PDIA3 interacts with calcitriol, an active form of vitamin D3, which could regulate gene expression through binding to the nuclear receptor vitamin D receptor (VDR) in cells [50]. Interestingly, genetic polymorphism of the VDR promoter, which controls VDR expression and T cell activation, is associated with the development of CIA [51]. Moreover, we could also show that the oxidized P4HB and PDIA3 protein levels were enhanced in day 7 CIA splenocytes from NCF4 [58A] mice.

APCs, including DCs and macrophages, are likely important in the pathogenesis of RA [52], but their role is dependent on subtype, location, and timing. Here we show that a modest effect on intracellular burst associated with a single amino acid replacement in NCF4, which has also been reported in humans, could in fact twist macrophages and DCs to enable priming of T cells into arthritogenicity. Importantly, RA is not only associated with NCF1 polymorphism [3,4] but is also associated with a haplotype containing the *NCF4* gene [2].

## 4 Material and methods

### 4.1 Mice and arthritis models

NCF4^A58/58R^ heterozygous mice (backcrossed to B10.Q > 10 generations) were bred within a specific pathogen-free (FELASA2) isolated unit within the animal department of Karolinska Institute, Stockholm). The experiments were reproduced under conventional conditions in the Xi'an Jiaotong University animal center. Male littermates were used for animal and cell experiments. The genotyping reagents are listed in Supplementary Table 1. All the mice experiments were approved by the regional animal ethics committee for animal research in Stockholm, Sweden (N35/16) or at Xi'an Jiaotong University (No. 2017-666 and 2020-1095). All animal experiments followed animal research experimental guideline ARRIVE [53], i.e. including age and cage matching, use of littermate offspring from fully backcrossed strains and mouse identity blinded for the investigator during experiments.

To induce GIA in mice, all the male mice over 3 months of age were given a single intradermal injection of 100 μL of emulsified hGPI_325-339_ peptide (10 μg per mouse) 1:1 complete Freund's adjuvant (CFA) (10 mg/mL *Mycobacterium tuberculosis* H37Ra (BD 231141) in incomplete Freund's adjuvant (IFA) (BD 263910)) as described previously [54]. To induce CIA in mice, all the mice over 3 months were given a single intradermal injection of 100 μL of emulsified type II collagen (50 μg per mouse) 1:1 CFA (BD, 263810) as described previously [31]. Arthritis severity was scored daily after immunization, as earlier described in detail [55]. In brief, one point was given for each inflamed knuckle or toe and up to five points were given for an affected ankle (in total 15 points/paw). Mice were sacrificed according to the experiment plans, including a collection of blood and tissues.

### 4.2 FTY720 treatment

FTY720 (from Aladdin, F126599) was dissolved in water to 1 mg/mL concentration in PBS. All the mice were weighed and then immunized with the hGPI_325-339_ peptide, then the mice were randomly assigned to PBS (A) or FTY720 (B) group. A or B (3.3 μg FTY720/g bodyweight) was injected intraperitoneally into mice daily according to each mouse's body weight.

### 4.3 Excision of iLN

Mice immunized with hGPI_325-339_ were used for excision of LNs at day 4, 7 and 8 after immunization. They were anaesthetized with isoflurane during the operation. An ophthalmic scissor was used to open a small incision on the skin near the inguinal region on one side, and then a tweezer was used to isolate the iLNs on both sides. Finally, clips were used to close the skin. The same procedure was applied to sham operated mice but without taking out the iLNs.

### 4.4 iLN cell transfer experiment

For cell transfer experiments, all the hGPI325-339 immunized mice were terminated on day 7. The iLNs were taken out, mashed on a 0.45 μm cell strainer, washed with PBS, and then lymphocytes were counted. Subsequently 5 × 10^7^ cells from either NCF4^R58^ or NCF4^58A^ were transferred by i.v. injection to immunized B10Q mice on day 7, immediately after the mice had their iLNs removed on both sides. All recipients were observed for development of arthritis.

### 4.5 Macrophage depletion experiment

To deplete macrophages, a previously used protocol was followed [56]. In brief, two days after immunization, the mice received a single intravenous injection (200 μl of clodronate liposome or PBS liposome). On day 8, all the mice received a second single intravenous injection. Detailed methods can be found in the supplementary data.

### 4.6 Histology and immunohistochemistry

Histologic analysis was performed using paraffin sections from the paws after staining with hematoxylin and eosin (H&E). The degree of synovitis was evaluated by four parameters: the thickness of synovial lining layers, pannus, synovial inflammatory cells, and angiogenesis (0–2 points for each parameter). Joint destruction was evaluated by the degree of cartilage erosion, bone erosion, joint ankle loss, and change of articular structure (0–2 points for each parameter) [57].

For immunohistochemistry staining we used 5 μm-thick tissue sections treated with 3% H_2_O_2_ for 20 min at room temperature, washed with PBS 3 times, and incubated overnight at 4 °C with the CXCR3 antibody (1:100) diluted in antibody dilution buffer (containing 0.1% of Triton X-100). The next morning, the samples were washed with PBS 3 times, and then incubated with the secondary antibody for 30 min, followed by the DAB kit and hematoxylin staining as usual. The images were captured by microscope (Olympus, Japan) and analyzed by the IPP6.0 software. Additional information for IHC is detailed in Supplementary Table 2.

### 4.7 Antibody analysis

All mice were anaesthetized by inhalation in a chamber, and then cheek bled to obtain 0.1 mL blood in marked EP tubes. The blood was kept at room temperature for 1 h, centrifuged at 3000 rpm for 10 min, and then serum was collected and stored at −20 °C. Serum was diluted to 1:1000 for anti-hG6PI analysis. ELISA MaxiSorp plates were coated with 50 μL of 10 μg/mL of the recombinant protein in PBS. The amounts of total IgG were determined through quantitative ELISA using peroxidase-conjugated goat anti-mouse IgG (H + L, 115-035-062, Jackson ImmunoResearch, USA) secondary antibody. ABTS (2,2′-azino-bis (3-ethylbenzthiazoline-6-sulfonic acid), #11204521001, Roche Diagnostics GmbH, Germany) was used as substrate. All the values of each well were measured at 405 nm and were expressed as optical density values. Detailed information for ELISA is presented in Supplementary Table 3.

### 4.8 Flow cytometry

Antibodies were purchased from Biolegend and BD Biosciences and included anti-CD45, anti-CD3, anti-CD4, anti-CD8, anti-CD11b, anti-CD11c, anti-F4/80, anti-Ly6G, anti- Ly6C, anti-CD44, anti-CD62L, anti-CXCR3, and anti-CCR6. Briefly, cells were isolated from iLNs, spleen, and peripheral blood, and then counted. All cells were first incubated with live and dead dye and FcR blocker (24G2 antibodies, BD, 553141) for 10 min, and then the mixture of antibodies was added and cells were stained for another 20 min, followed by washing with PBS 3 times. All cells were resuspended in 3% BSA PBS and then acquired on an LSRII flow cytometer. All data were analyzed by FlowJo software. Information on the antibodies used and concentration and dilution details are presented in Supplementary Table 4.

### 4.9 T cell recall assays

Peptides spanning the sequence 325–339 of human GPI (H-IWYINCFGCETHAML-OH) with c-c or modified s-s (H-IWYINSFGSETHAML-OH) were synthesized as previously described [23,24]. For the T cell recall assay, 5 × 10^5^ draining lymph node cells were plated per well in precoated ELISA plates. Plates were precoated with anti-IL2 (Jes6-1A12), anti-IFNγ (Mabtech, #3321-3-1000), or anti-IL17 (TC11-18H10.1) overnight, and washed with PBS. Then the cells were stimulated with c-c or s-s peptides for 48 h. The cytokines (IL2, IFNγ, and IL17) were detected with biotinylated anti-IL2 (Jes6-5H4), anti-IFNγ (Mabtech, #3321-6-1000), and anti-IL17(TC11-8H4) for 2 h, and then washed with PBS 4 times, followed by Eu^3+^ labeled streptavidin (PerkinElmer #1244-360). The Eu^3+^ labeled streptavidin was measured by dissociation-enhanced time resolved ﬂuorometry (excitation 360/40 and emission 620/40, Synergy-2, BioTek Instruments). All the values of each well were expressed as optical density values. Detailed information for ELISA is included in Supplementary Table 3.

### 4.10 T cell isolation and activation assay

Briefly, all the T cells were isolated following the protocol from Dynabeads™ Untouched™ Mouse T Cells Kit (Thermo fisher, 11413D). hGPI_325-339_ immunized mice were terminated on day 7. The iLNs were taken out, mashed on a 0.45 μm cell strainer, and washed with PBS. The lymphocytes from iLNs were collected and counted, adjusted to a concentration of 5 × 10^7^ cells in 500 μL isolation buffer, and then the antibody mix was added with inactivated FBS and incubated for 20 min at 4 °C. Cells were then washed with isolation buffer, resuspended in 4 mL isolation buffer with 1 mL pre-washed Dynabeads, and incubated for 15 min at room temperature, after which the supernatant was collected. The isolated cells were separated to check the purity or to activate T cells with 5 μg/mL anti-CD3 and 5 μg/mL anti-CD28 antibody, according to the directions. All the T cells were collected and lysed with RIPA buffer for T cell signal activation detection. The isolated cells were stained with anti-CD3 FITC antibody to check their purity. All the detailed information for T cell isolation and activation is in Supplementary Table 5.

### 4.11 Antigen presentation assay

For antigen presentation assays, 1 × 10^5^ splenocytes were co-cultured with 1 × 10^5^ G5 T cell hybridoma [24] for 24 h in 96 U type well plates in the presence of GPI with c-c peptide or modified s-s peptide. Peptides were synthesized and purified (>95%) by Biomatik (Wilmington, DE, USA); the sequences were the same as reported by Yang et al. [24]. IL-2 in the culture supernatant was detected by ELISA using Jes6-1A12 (2 μg/mL) capture antibody, biotinylated Jes6-5H4 (1 μg/mL) detection antibody, and Eu-labeled streptavidin (PerkinElmer). The Eu^3+^label was measured by dissociation-enhanced time-resolved ﬂuorometry (excitation 360/40 and emission 620/40, Synergy-2, BioTek Instruments). Detailed information for ELISA is in Supplementary Table 3.

### 4.12 ROS detection

The total, extracellular, and intracellular ROS were detected by luminol- and isoluminol-based chemiluminescence assays [11]. Intracellular ROS for different cell types was detected by flow cytometry. Briefly, cells were first stained with cell surface markers, incubated with 3 μM of DHR123 in DMEM for 10 min and then stimulated with 200 nM PMA for 20 min in a 37 °C cell incubator. Then all the cells were washed with PBS 3 times, detected by flow cytometry for different cell types, and the MFI of DHR was calculated for statistics. Detailed information for ROS detection is presented in Supplementary Tables 4 and 6.

### 4.13 BMM and BMDC

Briefly, bone marrow cells were isolated from NCF4^R58A^ and NCF4^WT^ mice and seeded at the density of 2 × 10^6^ cells per mL in RPMI 1640 with 10% FBS and 20 ng/ml M-CSF for differentiating bone marrow–derived macrophages (BMM) [58] or RPMI 1640 with 10% FBS and 20 ng/ml GM-CSF and 10 ng/mL IL-4 for differentiating bone marrow–derived dendritic cells (BMDC) [59]. To induce classical activated macrophages, IFNγ 20 ng/mL and LPS 100 ng/mL were used [60]. Detailed information for BMM/BMDC and CAM is in Supplementary Table 7.

### 4.14 Western blot (WB)

Total protein lyses from T cell and macrophages were extracted by the RIPA (P0013C, Beyotime, China) solution with a mixture of protease and phosphatase inhibitors (5892970001, 4906845001 Merck). The protein concentration of each sample was determined by a BCA kit (23227, Thermo). One tablet of protease inhibitor was dissolved in 50 mL RIPA solution, and one tablet of phosphatase inhibitor was dissolved in 10 mL RIPA solution. All the protein samples were added with 5 × loading buffer and heated at 95 °C for 10 min. Total proteins (20 μg) for each sample were separated by 4–10% SDS-PAGE gels according to standard procedures with the Bio-Rad system. The primary antibody was applied and incubated at 4 °C overnight; primary antibodies included anti-*p*-SRC, anti-SRC, anti-p-ZAP70, anti-ZAP70, anti-*p*-LAT, anti-β-ACTIN, anti-NCF1, anti-NCF2, anti-NCF4, anti-Nox2 and anti-H3. The signal was further detected using the secondary antibodies of goat anti-rabbit/mouse IgG conjugated with HRP. Signal intensity was determined by Supersignal West Pico Kit. Data are shown with representative images and the density of the bands was measured by ImageJ software and normalized to β-actin, H3 histone, or its own total protein. Detailed information for WB is in Supplementary Tables 8-1 and 8-2.

### 4.15 Redox WB

All the splenocytes from the GIA or CIA models were lysed with 100 mM N-ethylmaleimide (NEM) in RIPA (with protease inhibitor) 1 h on ice [14]. The NEM linked to the free thiol group and increased the protein molecular weight. The principle of NEM linked Redox WB is shown in S. Fig. 4. The positive controls are cells treated with 5 mM of β-ME or 5 mM of H_2_O_2_, respectively. The protein concentration was determined by BCA kits. The protein samples were added to 5 × non-reducing loading buffer and boiled at 95 °C for 10min. The samples were separated by 8%, 10% or 12.5% SDS-PAGE gels, and then transferred to PVDF membrane using a Bio-Rad system. The primary antibodies (anti-GILT, anti- PDI, and anti-PDAI3) were applied at 4 °C overnight. The signal was further detected by using the secondary antibodies of goat anti-rabbit/mouse IgG conjugated with HRP. Signal intensity was determined by Supersignal West Pico Kit. Data were expressed by showing one representative image, and the density of the bands was measured by ImageJ software. The ratio of Ox-form/Red-form was calculated. Detailed information for WB is in Supplementary Tables 8-1 and 8-2.

### 4.16 RNA isolation and RT-qPCR

Total RNA from the splenocytes and cells was lysed with TRI Reagent® and reverse transcribed to cDNA using the First Strand cDNA Synthesis Kit according to the manufacturers’ protocol (K1622, Thermo scientific). qPCR was performed by using iQ5 optical system with SYBR green for real time quantification of the relative gene expression. The primers used for the gene primers are listed in Supplementary Table 9. Gene expression analyses were normalized with *Actb* with 2^−ΔΔCt^. Detailed information on the analyses is presented in Supplementary Table 9.

### 4.17 RNA-seq and analysis

BMMs from NCF4^R58^ and NCF4^58A^ were activated as classical activated macrophages for 6 h, and the cells were collected with TRIzol. Isolated T cells were co-cultured with macrophages from NCF4^R58^ and NCF4^58A^ stimulated with GPI_325-339_ peptide for 24 h and then collected with TRIzol. All the RNA sequencing services were performed by Novogene Company, China.

Total RNA was extracted using TRIzol. The quality of all RNA samples was evaluated using RNA electrophoresis and Nano Photometer®spectrophotometer (IMPLEN, CA, USA). mRNA was isolated by Oligo Magnetic Beads and cut into small fragments for cDNA synthesis. Libraries were generated using the NEBNext UltraTM RNA Library Prep Kit (New England Biolabs, Ipswich, MA, USA) for the Illumina system following the manufacturer's instructions. Sequencing was conducted using the Illumina Hiseq XTEN platform.

The differentially expressed mRNAs were selected if they had a fold change >2 or a fold change <0.5 and a p value < 0.05, as determined by R package edgeR or DESeq2, and were then analyzed with GO and KEGG enrichment of the differentially expressed mRNAs. Gene set enrichment analysis (GSEA) in differentially expressed mRNAs was also conducted to explore the KEGG pathways associated with the hub genes using the ‘ClusterProfiler’ R package. For all comparisons, an FDR-adjusted p value of 0.05, was considered to be the threshold for statistical significance with application of the Benjamini-Hochberg test for multiple testing correction.

### 4.18 Statistics

Data were expressed as mean ± SD and analyzed using SPSS software. The Shapiro-Wilk test was employed to validate the normal distribution of data. Statistical analysis was performed by one-way ANOVA among groups, and the Student t-test was employed to analyze the signiﬁcant differences between two groups. The Mann-Whitney *U* test was used to analyze the density of Western blotting bands. The Multiple *t*-test with Holm-Sidak's comparison correction was used to analyze the clinical score of arthritis. The Kruskal–Wallis test with Dunn's *post hoc* test was used to analyze ELISA data. Statistical signiﬁcance levels in figures are expressed as *p﹤0.05, **p﹤ 0.01, ***p﹤0.001, and****p﹤0.0001.

## CRediT authorship contribution statement

**Jing Xu:** Writing – review & editing, Writing – original draft, Project administration, Investigation, Conceptualization. **Chang He:** Writing – review & editing, Investigation. **Yongsong Cai:** Investigation. **Xipeng Wang:** Writing – review & editing, Methodology, Investigation. **Jidong Yan:** Methodology. **Jing Zhang:** Methodology. **Fujun Zhang:** Methodology. **Vilma Urbonaviciute:** Writing – review & editing, Methodology. **Yuanyuan Cheng:** Methodology. **Shemin Lu:** Supervision. **Rikard Holmdahl:** Writing – review & editing, Supervision.

## Declaration of competing interest

The authors declare no competing financial interests.

## Data Availability

Data will be made available on request.
