# Supplementary material for: NCF4 regulates antigen presentation of cysteine peptides by intracellular oxidative response and restricts activation of autoreactive and arthritogenic T cells
Source: Redox Biol. 2024 Mar 26;72:103132. doi: 10.1016/j.redox.2024.103132 (PMC11096609; doi:10.1016/j.redox.2024.103132)

Supplementary Table 1 Reagents for NCF4 genotype and GIA model

|  | Reagents and Resource | | Suppliers/ Identifier |  |
| --- | --- | --- | --- | --- |
| Ncf4^58A^ mutation genotype | TaqMan Master mix | | Thermo, 4369016 |  |
| primers and probes | Ncf4_MUT | FAM-TACCGCGCCTATC | Sangong Biotech |  |
|  | Ncf4_W T | VIC-ACCGCCGCTATC |  |  |
|  | Ncf4_F | CAAAAGGAGGGTCCAAGTATCTCA |  |  |
|  | Ncf4_R | CAAACCGCTCCTCGAGCTT |  |  |
| Animal model | hGPI_325-339_(c-c) (dissolved in DMSO for 10mg/ml) | | Biomatik, or Sangong Biotech | 5mg |
|  | IFA | | BD, 263910 | 10 X 10ml |
|  | CII | | home made |  |
|  | CFA | | BD, 263810 | 10 X 10ml |
|  | Mycobacterium tuberculosis H37Ra | | BD, 231141 | 6 X 100mg |
|  | Clodronate liposomes and control liposones | | LIPOSOMA, CP-005-005 | 5 ml |
|  | FTY720 | | Aladdin, F126599 | 1g |
|  | Isoflurane | | Baxter, KDG9623 |  |

Supplementary Table 2 Reagents for CXCR3 IHC

|  | Reagents and Resource | Suppliers/ Identifier | |  | |
| --- | --- | --- | --- | --- | --- |
| IHC | CXCR3 antibody (IHC, 1:200 diluted, 4℃, O/N) | Proteintech 26756-1-AP | 750μg/ml | |  |
|  | DAB kit | Boster, AR1027 | |  | |
|  | SABC-POD(F) Rabbit | Boster, SA1028 | |  | |

Supplementary Table 3 Reagents for T cell recall, antigen processing assay

|  | Reagents and Resource | Suppliers/ Identifier |  |
| --- | --- | --- | --- |
| ELISA \ T cell recall \ Antigen processing assay | hGPI protein | home made |  |
|  | peroxidase-conjugated goat anti- mouse IgG (H+L) | Jackson ImmunoResearch, 115-035-062 |  |
|  | ABTS | Roche, 11204521001 | 1mg |
|  | ABTS Buffer 10X | Roche, 11112597001 |  |
|  | hGPI_325-339_(s-s) (desolved in DMSO for 10mg/ml) | Biomatik | 5mg |
|  | Cap-IL2 antibody | Jes6-1A12, home made |  |
|  | Det-IL2 antibody | Jes6-5H4, home made |  |
|  | Cap-IL17 antibody | TC11-18H10.1, home made |  |
|  | Det-IL17 antibody | TC11-8H4, home made |  |
|  | Cap-IFNG antibody | Mabtech, #3321-3-1000 |  |
|  | Det-IFNG antibody | Mabtech, #3321-6-1000 |  |
|  | Eu3+ label streptavidin | PerkinElmer, 1244-360 |  |
|  | DELFIA® Enhancement Solution | PerkinElmer, 1244-105 |  |
|  | Tween-20 | Sigma, P1379 | 1L |
|  | PBS | home made |  |

Supplementary Table 4 Reagents for FACS

|  | Reagents and Resource | | Suppliers/ Identifier |  |
| --- | --- | --- | --- | --- |
| Synovial cell isolation enzymes | DNaseI | final concentration 0.2mg/ml | Roche, 04716728001 |  |
|  | Collagenase IV | final concentration 1mg/ml | Yeasen Biotechnology, 40510ES60 | 100mg |
| Staining fixation permeabilization buffers | BD Cytofix/Cytoperm™ | | BD, 554714 |  |
|  | BD transcription factor buffer set | | BD,562574 |  |
| L/D dye | Yellow fluorescent reactive dye (FACS, 1:1000 diluted) | | Invitrogen, 2438368 |  |
|  | LIVE/DEAD near-IR (FACS, 1:1000 diluted) | | Invitrogen, L34976 |  |
| FACS antibodies | CD16/CD32 antibody (10μg/ml in FACS buffer) | | BD, 553142 | 0.5 mg/ml |
|  | APC-CD45 (FACS, 1:200 diluted, 4℃, 30 min) | | BD, 559864 | 0.1 mg/ml |
|  | Percp.Cy5.5-CD3 (FACS, 1:200 diluted, 4℃, 30 min) | | Biolegend, 152312 | 0.2 mg/ml |
|  | BV650-CD4 (FACS, 1:200 diluted, 4℃, 30 min) | | BD, 563747 | 0.2 mg/ml |
|  | PE-CD44 (FACS, 1:200 diluted, 4℃, 30 min) | | BD, 553134 | 0.2 mg/ml |
|  | FITC-CD3 (FACS, 1:200 diluted, 4℃, 30 min) | | BD, 561798 | 0.5 mg/ml |
|  | PE.Cy7-CCR6 (FACS, 1:200 diluted, 4℃, 30 min) | | Biolegend, 129816 | 0.2 mg/ml |
|  | PE-CD25 (FACS, 1:200 diluted, 4℃, 30 min) | | BD, 553866 | 0.2 mg/ml |
|  | PE-CCR6 (FACS, 1:200 diluted, 4℃, 30 min) | | Biolegend, 129803 | 0.2 mg/ml |
|  | APC-CXCR3 (FACS, 1:200 diluted, 4℃, 30 min) | | BD, 562266 | 0.2 mg/ml |
|  | A647-Foxp3 (FACS, 1:200 diluted in perm/wash, 4℃, 50 min) | | BD, 563486 | 0.2 mg/ml |
|  | PE.Cy5-CD4 (FACS, 1:200 diluted, 4℃, 30 min) | | Biolegend, 100514 | 0.2 mg/ml |
|  | FITC-F4/80 (FACS, 1:200 diluted, 4℃, 30 min) | | Biolegend, 123108 | 0.5 mg/ml |
|  | BV711-F4/80 (FACS, 1:200 diluted, 4℃, 30 min) | | Biolegend, 123147 | 0.2 mg/ml |
|  | BV785-CD45.2 (FACS, 1:200 diluted, 4℃, 30 min) | | Biolegend, 109839 | 0.2 mg/ml |
|  | APC-CD11b (FACS, 1:200 diluted, 4℃, 30 min) | | Biolegend, 101212 | 0.2 mg/ml |
|  | APC.Cy7-CD11c (FACS, 1:200 diluted, 4℃, 30 min) | | Biolegend, 117324 | 0.2 mg/ml |
|  | PB-Ly6G (FACS, 1:200 diluted, 4℃, 30 min) | | Biolegend, 127612 | 0.5 mg/ml |
|  | PE.Cy7-B220 (FACS, 1:200 diluted, 4℃, 30 min) | | Biolegend, 103222 | 0.2 mg/ml |
|  | APC.Cy7-TCRb (FACS, 1:200 diluted, 4℃, 30 min) | | Biolegend, 109220 | 0.2 mg/ml |
|  | AF700-TCRb (FACS, 1:200 diluted, 4℃, 30 min) | | Biolegend, 109224 | 0.5 mg/ml |
|  | AF700-MHC IA/IE (FACS, 1:200 diluted, 4℃, 30 min) | | Biolegend, 107622 | 0.5 mg/ml |
|  | BV650-F4/80 (FACS, 1:200 diluted, 4℃, 30 min) | | BD, 743282 | 0.2 mg/ml |
|  | AF700-IL17A (FACS, 1:200 diluted in perm/wash, 4℃, 50 min) | | BD, 560820 | 0.2 mg/ml |
|  | PB-IFNG (FACS, 1:200 diluted in perm/wash, 4℃, 50 min) | | Biolegend, 505818 | 0.5 mg/ml |
|  | PB-CD11b (FACS, 1:200 diluted, 4℃, 30 min) | | Biolegend, 101224 | 0.5 mg/ml |
|  | PE-CD11c (FACS, 1:200 diluted, 4℃, 30 min) | | Biolegend, 117307 | 0.2 mg/ml |
|  | FITC-Ly6C (FACS, 1:200 diluted, 4℃, 30 min) | | BD, 553104 | 0.5 mg/ml |
|  | APC-F4/80 (FACS, 1:200 diluted, 4℃, 30 min) | | Biolegend, 123116 | 0.2 mg/ml |

Supplementary Table 5 Reagents for T cell activation

|  | Reagents and Resource | Suppliers/ Identifier |  |
| --- | --- | --- | --- |
| T cell activation | CD3ε antibody | BD, 553058 | 0.5 mg/ml |
|  | CD28 antibody | BD, 553294 | 1 mg/ml |
|  | Untouched T cell isolated kit | Invitrogen, 11413D |  |

Supplementary Table 6 Reagents for ROS detection

|  | Reagents and Resource | Suppliers/ Identifier |  |
| --- | --- | --- | --- |
| ROS detection | DHR-123 | Aladdin, D115501 | 5mg |
|  | PMA | Aladdin, P167764 | 1mg |
|  | Luminol | Sigma, A8511 | 5g |
|  | Isoluminol | Tokyo Chemical Industry, 3682-14-2 | 1g |
|  | SOD | Beyotime, S0086 |  |
|  | HBSS | Beyotime, C0219 |  |
|  | Peroxidase | Sigma, P8250 | 5KU |
|  | fMLF | Sigma, F3506 | 10mg |
|  | Catalase | Beyotime, S0082 |  |

Supplementary Table 7 Reagents for BMDM, BMDC and CAM

|  | Reagents and Resource | Suppliers/ Identifier |
| --- | --- | --- |
| BMM/BMDC inducer | MCSF final concentration 20ng/ml | Peprotech, 315-02 |
|  | GMCSF final concentration 20ng/ml | Peprotech, 315-03 |
|  | IL4 final concentration 10ng/ml | Peprotech, 214-14 |
| Classical activation macrophage inducer | IFNG final concentration 20ng/ml | home made |
|  | LPS final concentration 100ng/ml | Sigma, L2630 |

Supplementary Table 8-1 Reagents for Redox WB and WB

|  | Reagents and Resource | Suppliers/ Identifier |
| --- | --- | --- |
| WB and Redox WB | Proteinase inhibitor | Merck, 5892970001 |
|  | phosphate inhibitor | Merck, 4906845001 |
|  | RIPA | Beyotime, P0013C |
|  | NEM | MCE, HY-D0843 |
|  | DTT | MCE, HY-15917 |
|  | H_2_O_2_ | Aladdin, H433859 |
|  | SuperSignal™ West Pico PLUS | Thermo, 34580 |
|  | BCA kit | Thermo, 23227 |

Supplementary Table 8-2 Reagents for Redox WB and WB antibodies

|  | Reagents and Resource | Suppliers/ Identifier |  |
| --- | --- | --- | --- |
| WB and Redox WB Antibodies | p-SRC (WB, 1:1000 diluted, 4℃, O/N) | CST, 2101 | 734 µg/ml |
|  | SRC (WB, 1:1000 diluted, 4℃, O/N) | CST, 2109 | 139 µg/ml |
|  | p-ZAP70 (WB, 1:1000 diluted, 4℃, O/N) | CST, 2704 | 55 µg/ml |
|  | ZAP70 (WB, 1:1000 diluted, 4℃, O/N) | CST, 2705 | 142 µg/ml |
|  | p-LAT (WB, 1:1000 diluted, 4℃, O/N) | CST, 54364 | 100 µg/ml |
|  | p-P65 (WB, 1:1000 diluted, 4℃, O/N) | CST, 3033 | 57 µg/ml |
|  | P65 (WB, 1:1000 diluted, 4℃, O/N) | CST, 8242 | 208 µg/ml |
|  | p-STAT1 (WB, 1:1000 diluted, 4℃, O/N) | CST, 7649 | 42 µg/ml |
|  | STAT1 (WB, 1:1000 diluted, 4℃, O/N) | CST, 14994 | 53 µg/ml |
|  | p-STAT3 (WB, 1:1000 diluted, 4℃, O/N) | CST, 9145 | 100 µg/ml |
|  | STAT3 (WB, 1:1000 diluted, 4℃, O/N) | CST, 9139 | 44 µg/ml |
|  | Actinb (WB, 1:1000 diluted, 4℃, O/N) | CST, 4970 | 61 µg/ml |
|  | NCF1 (WB, 1:1000 diluted, 4℃, O/N) | Invitrogen, PA5-31169 | 1mg/ml |
|  | NCF2 (WB, 1:1000 diluted, 4℃, O/N) | BD, 610912 | 250μg/ml |
|  | NCF4 (WB, 1:1000 diluted, 4℃, O/N) | Invitrogen, PA5-75191 | 1mg/ml |
|  | NOX2 (WB, 1:1000 diluted, 4℃, O/N) | BD, 611414 | 250μg/ml |
|  | Vinculin (WB, 1:1000 diluted RT, 2h) | Proteintech, 66305-1-Ig | 1mg/ml |
|  | H2B (WB, 1:1000 diluted, 4℃, O/N) | CST, 4499 | 10 µg/ml |
|  | PDIA3 (WB, 1:1000 diluted, RT, 2h) | Proteintech, 15967-1-AP | 550 μg/ml |
|  | GILT (WB, 1:200 diluted, 4℃, O/N) | Bioss Inc. bs-13591R | 1mg/ml |
|  | P4HB (WB, 1:1000 diluted RT, 2h) | Proteintech, 11245-1-AP | 850 μg/ml |

Supplementary Table 9 Reagents for RNA isolation and QPCR

|  | Reagents and Resource | | Suppliers/ Identifier |
| --- | --- | --- | --- |
| RNA isolation and QPCR | TRI Reagent® | | Sigma, 93289 |
|  | cDNA synthesis kit | | Thermo, K1622 |
|  | SybrGreenFastStart Universal SYBR Green Master (Rox) | | Roche, 4913914001 |
| primers | Mus_Stat1 F | TCACAGTGGTTCGAGCTTCAG | Sangong Biotech |
|  | Mus_Stat1 R | CGAGACATCATAGGCAGCGTG |  |
|  | Mus_Ifna F | TTCCCCTGACCCAGGAAGATG |  |
|  | Mus_Ifna R | TCTCTCAGTCTTCCCAGCACATT |  |
|  | Mus_Tnfa F | ACGTCGTAGCAAACCACCAA |  |
|  | Mus_Tnfa R | ATAGCAAATCGGCTGACGGT |  |
|  | Mus_Il1b F | CTGTGTCTTTCCCGTGGACCT |  |
|  | Mus_Il1b R | TTGTTGTTCATCTCGGAGCCT |  |
|  | Mus_iNos F | TATGCTGTGTTTGGCCTTGG |  |
|  | Mus_iNos R | GCGGCTGGACTTTTCACTCT |  |
|  | Mus_Il6 F | GAGGATACCACTCCCAACAGACC |  |
|  | Mus_Il6 R | AAGTGCATCATCGTTGTTCATACA |  |
|  | Mus_Pdia3 F | CGCCTCCGATGTGTTGGAA |  |
|  | Mus_Pdia3 R | CAGTGCAATCCACCTTTGCTAA |  |
|  | Mus_Erap1 F | TAATGGAGACTCATTCCCTTGGA |  |
|  | Mus_Erap1 R | AAAGTCAGAGTGCTGAGGTTTG |  |
|  | Mus_Ifi30 F | CCTGGTCTCCGATCCTACCAT |  |
|  | Mus_Ifi30 R | TTGCAGGTGGTTGTGCCTT |  |
|  | Mus_P4hb F | GCCGCAAAACTGAAGGCAG |  |
|  | Mus_P4hb R | GGTAGCCACGGACACCATAC |  |
|  | Mus_Pdia4 F | GGCTGACAAAGATACAGTGCT |  |
|  | Mus_Pdia4 R | GTGGGGTAGCCACTCACAT |  |
|  | Mus_Pdia6 F | TCCAGGGATTTCCTACCATCAA |  |
|  | Mus_Pdia6 R | GTCAGCTCTACCACGTCTTTC |  |
|  | Mus_Actb F | GGCTGTATTCCCCTCCATCG |  |
|  | Mus_Actb R | CCAGTTGGTAACAATGCCA TGT |  |

Supplementary Fig.1. T cell activation signal in NCF4^R58^ and NCF4^58A^


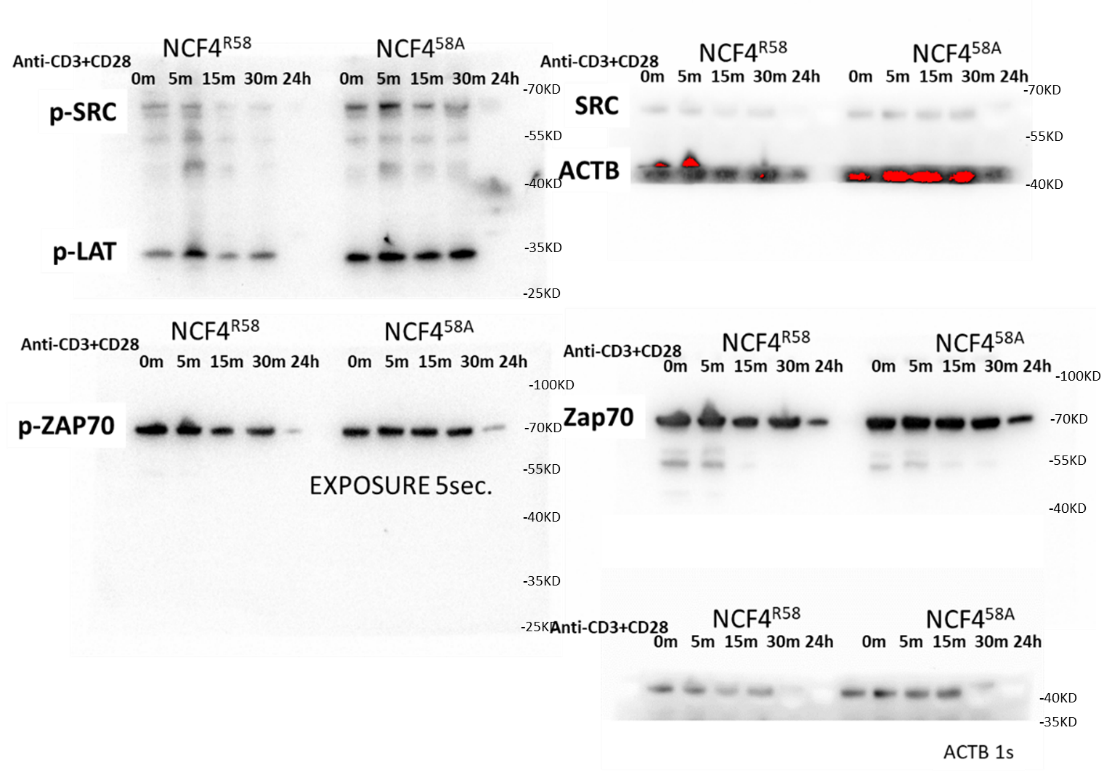


The original WB pictures for T cell activation signaling were listed in complete form.

p-SRC/SRC (~MW 60KD); p-ZAP70/ZAP70 (~MW 70KD); p-LAT (~MW 36/38KD); ACTB (~MW 42KD). “→” points to the bands for each protein.

Supplementary Fig.2. NOX2 complex related protein expression in macrophages and T cells


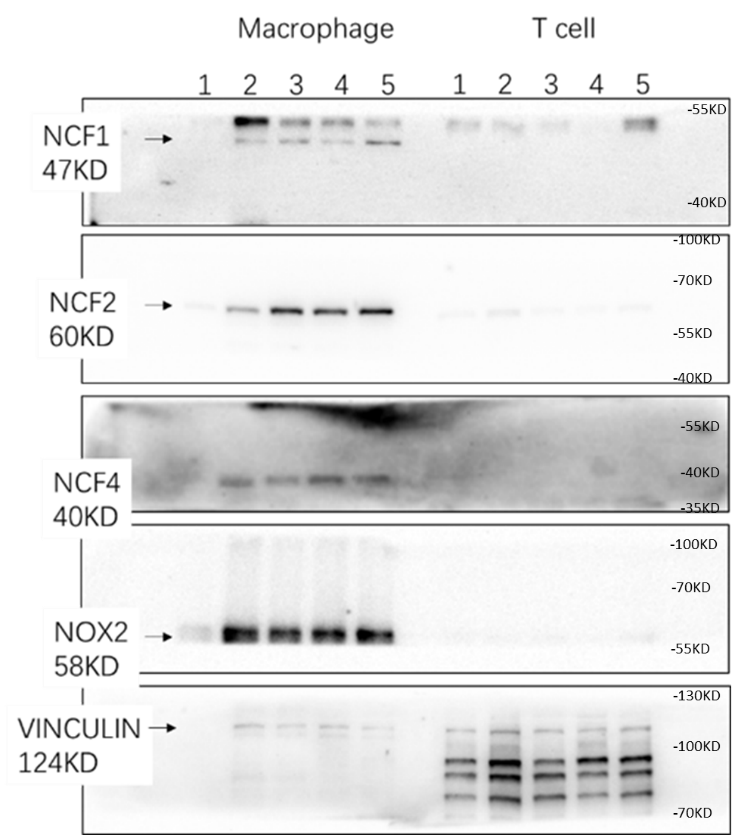


The original WB pictures for NOX2 complex related protein in macrophages and T cells were listed in complete form.

NCF1 (~MW 47KD); NCF2 (~MW 60KD); NCF4 (~MW 40KD); NOX2 (~MW 58KD); VINCULIN (~MW 124KD). “→” points to the bands for each protein.

Supplementary Fig.3. CAM activation signal in NCF4^R58^ and NCF4^58A^


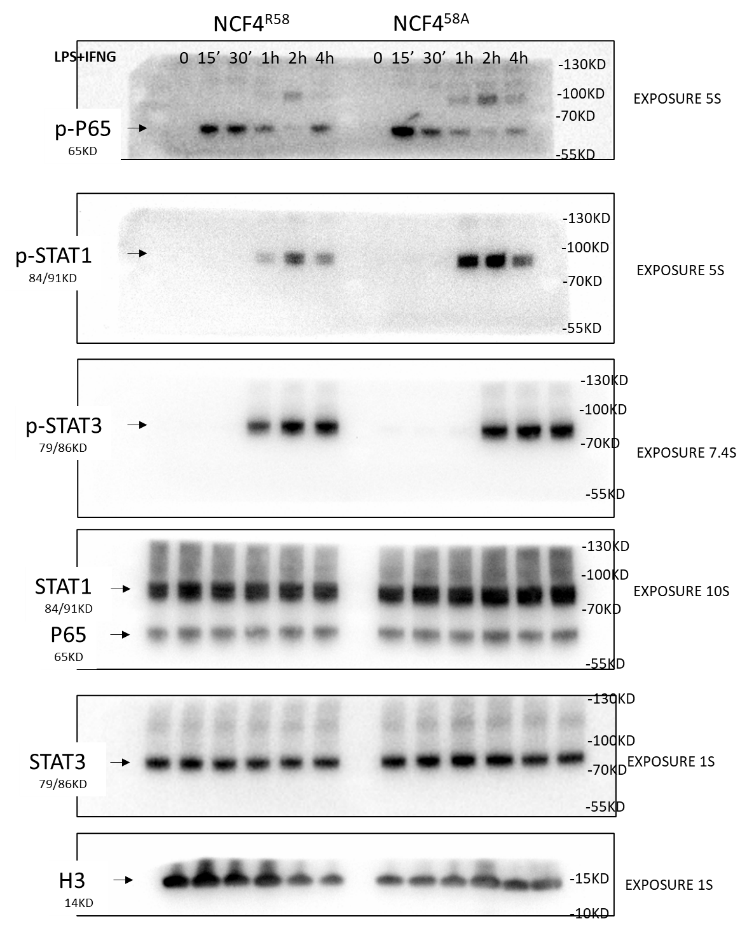


The original WB pictures for CAM activation signaling in macrophages were listed in complete form.

p-P65/P65 (~MW 65KD); p-STAT1/STAT1 (~MW 84/91KD); p-STAT3/STAT3 (~MW 79/86KD); H3 (~MW14KD). “→” points to the bands for each protein.

**Supplementary Methods**

**Isoluminol/ Luminol Assay (Total, Intracellular/ Extracellular ROS)**

1. Adjust cell concentration to 5 X 10^6^ cell/ml in HBSS (with Ca^2+^ and Mg^2+^)
2. Prepare 2X buffer as followed:

| Material | Isoluminoil buffer (Extracellular) | Luminol -ic buffer (Intracellular) | Luminol -Total buffer (Total) |
| --- | --- | --- | --- |
|  |  |  |  |
| HBSS (with Ca^2+^ and Mg^2+^) | 930μl | 915μl | 930μl |
| Iso/luminol (10mg/ml) | 35μl | 35μl | 35μl |
| HRPII (100U/ml) | 35μl |  | 35μl |
| Catalase (6.8X10^5^U/ml) |  | 30μl |  |
| SOD (3000U/ml) |  | 20μl |  |

1. Dilute 2X PMA (60ng/ml) in the buffer, add 50μL of PMA to each well
2. Start up the reader so it can heat up in time
3. Open the program and select new experiment
4. Chose the luminol assay for ox-burst protocol
5. Add 50μl of cell to a white assay plate
6. Add 50μ of buffers to the white assay plate using multichannel pipette.
7. Immediately start reading.

**Oxidative burst assay (FACS)**

1. Transfer the cells to a 96 well plate with V bottom
2. Prepare the antibodies for staining:

Anti-CD11b-APC

Anti-Ly6G-PB

Anti-CD11c-APC.Cy7

Anti-F4/80-BV711

- 1. (Do not use any of the fluorochromes: FITC, Alexa488, PE, PE-TexasRed, PE.Cy5, or PerCP, PerCP.Cy5.5)

1. Only for washed cells: stain the cell with FcR blocker (10μg/ml) for 10 min at RT.
2. Add prepared antibodies, incubate for 20 min at RT.
3. Lyse the red blood cells: 200μl per well of lysis buffer, spin it down, and wash once with PBS
4. Add 200μl of DMEM to the wells, add 25μl of 3μM DHR123 to the wells, incubate for 10 min for 37℃ 5% CO_2_ incubator
5. Add 25μl oh 200ng/ml PMA or control media to the wells, incubate for 20 min for 37℃ 5% CO_2_ incubator
6. Spin it down, and wash twice with PBS
7. Resuspend cells in PBS
8. Run with PMA stimulated first on the flow cytometer.

**Supplementary Methods:**

**Isolation of iLNs,** **lymphocytes, splenocytes cells**

1. Preparation of inguinal lymph nodes, spleen from immunized or naïve mice.
2. Transfer inguinal lymph nodes or spleen into a sterile mortar, and add 5ml PBS or 1640.
3. Remove the excessive fat and connective tissue by scissors.
4. Cut the inguinal lymph nodes or spleen to small pieces of approx.1-3mm size by scissors.
5. Using a 5ml pipette, thoroughly pipette up and down to gently wash the lymphocytes or splenocytes.
6. Filter lymphocytes or splenocytes-containing medium through a 100 μm strainer into a sterile 50 mL tube
7. Mesh the tissue with the accessories, add 5-10ml PBS or 1640 to the strainer.
8. Rinse cell strainer with fresh cell culture medium to fill up the tube to 30 mL.
9. Centrifuge the tube at 350g for 5min at RT.
10. Carefully discard the supernatant.
11. Gently resuspend the pellet in 10 mL cell culture medium and pass cell suspension through 70 μm strainer.
12. Centrifuge the tube at 350g for 5min at RT.
13. Resuspend cells in cell culture medium.
14. Using the cell counter to read the cell number.

For the splenocytes,

13’. Gently resuspend the pellet in 3 mL red blood cell lysis buffer and incubate in RT for 5 min.

14’. Centrifuge the tube at 350g for 5min at RT.

15’. Resuspend cells in cell culture medium.

16’. Using the cell counter to read the cell number.

**Supplementary Methods:**

Supplementary Fig.4. Principle of Redox WB


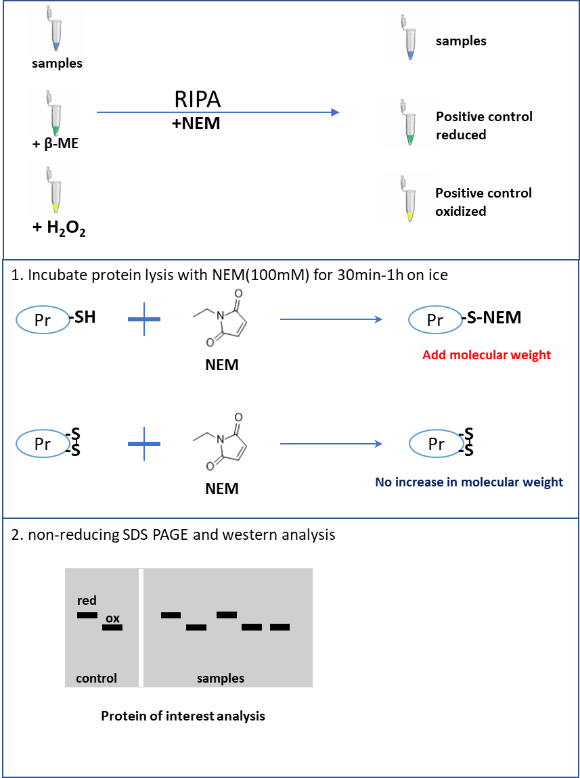

Supplement: Multimedia component 1 [file mmc1.docx]
